# Atraumatic acute compartment syndrome in anticoagulated patient: A case report

**DOI:** 10.1016/j.amsu.2022.104530

**Published:** 2022-09-02

**Authors:** Dara Ninggar Ghassani, Denny Suwanto, Meity Ardiana

**Affiliations:** Department of Cardiology and Vascular Medicine, Faculty of Medicine Universitas Airlangga – Dr. Soetomo General Academic Hospital, Jl. Mayjend Prof. Dr. Moestopo No 4-6, Surabaya, East Java, 60286, Indonesia

**Keywords:** Atraumatic, Acute compartment syndrome, Systemic anticoagulation, Case report

## Abstract

**Introduction and importance:**

Compartment syndrome is a well-known surgical emergency caused by increasing pressure inside the fascial or osteo-fascial compartment, resulting in vascular compromise, ischemia, and necrosis. This condition usually occurs following a traumatic incident. Here we present a report of nontraumatic acute compartment syndrome caused by systemic anticoagulation in patients presenting with the acute coronary syndrome.

**Case presentation:**

We report a case of a 51-year-old male with acute coronary syndrome receiving systemic anticoagulation, which later developed significant swelling and tensing on his right arm. He also complained of pallor and paresthesia with decreased peripheral oxygen saturation on his right arm.

**Clinical discussion:**

The patient was diagnosed with atraumatic acute compartment syndrome and underwent fasciotomy promptly. His symptoms improved after undergoing fasciotomy.

**Conclusions:**

Atraumatic acute compartment syndrome is a rare case. Identifying this condition without a typical history of underlying predisposition is important to avoid delaying emergent surgery as the key therapy.

## Highlights

• There are limited reports on the compartment syndrome of atraumatic origin.
• Anticoagulation and conditions that impairs coagulation may increase the risk of these hypothetical patophysiology
• Atraumatic acute compartment syndrome is a challenging diagnosis as the precipitating elements are not widely known.
• These cases can very easily be neglected and prompt treatment could be delayed.

## 1 Introduction and importance

Compartment syndrome is a well-known surgical emergency due to increased pressure within a fascial or osteo-fascial compartment, causing vascular compromise, and leading to ischemia and necrosis [[1], [2], [3]]. Acute compartment syndrome (ACS) generally occurs following a traumatic incident, but spontaneous compartment syndrome (without predisposing event), while infrequent, can also happen [[4], [5], [6]]. In addition, compartment syndrome occurring spontaneously as a complication of systemic anticoagulation is rare [6]. Few reports of acute compartment syndrome occurring spontaneously are published in the literature. A systematic review in 2019 showed that only 16 reports of spontaneous atraumatic ACS of the upper extremity were published from 1993 to 2016 [7]. Quick identification and decompression in this setting are crucial to prevent irreversible damage. This case report has been reported in line with the SCARE Criteria [8].

## 2 Case presentation

**Fig. 1:**
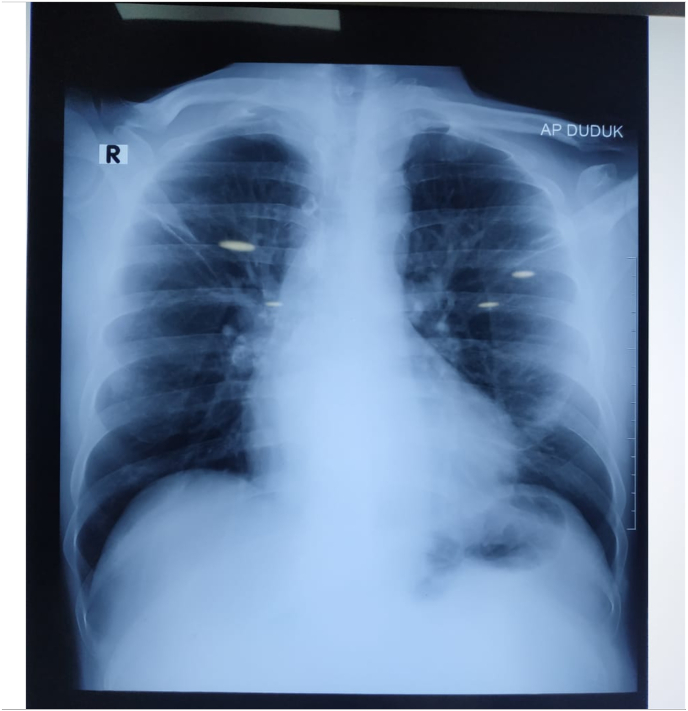
Normal chest X-Ray.

**Fig. 2:**
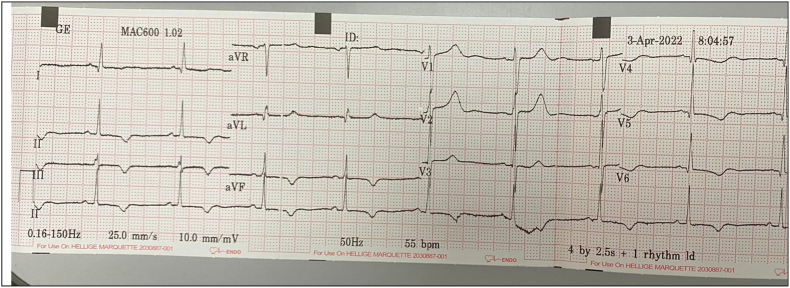
ECG showed ischemia at inferior and lateral leads.

We report a case of a 51-year-old male with crescendo chest pain presented at the referral hospital. He was previously diagnosed with chronic coronary syndrome and hypertension but refused coronary intervention. Otherwise, VAS (visual analog scale) of 8/10, vital signs and physical exam were within normal limit. Chest X-ray within normal limit (Fig. 1). There was T wave inversion at the inferior and lateral lead on his ECG (Fig. 2). Laboratory work showed an elevated serum creatinine level of 8.13 mg/dL and a significant elevation of HsTrop 289.6 ng/L (over 99 th percentile). He was then diagnosed as NSTEACS high risk and treated with double antiplatelet in loading dose (300 mg of aspirin and 300 mg of clopidogrel 300 mg) and enoxaparin 60 mg twice daily despite significant reduced estimated glomerular filtration rate of 9 ml/min.m2. After 3 days of care with nonsignificant improvement, he was referred to our hospital for further intervention.

**Fig. 3:**
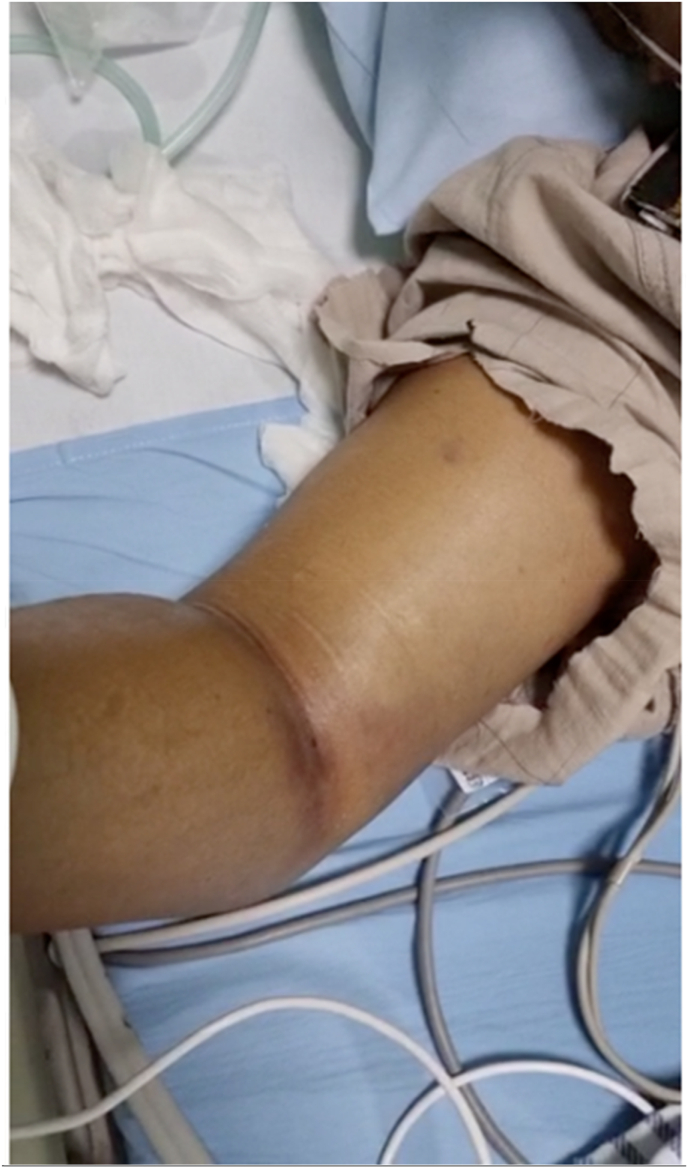
Swollen and tensing right arm.

**Fig. 4:**
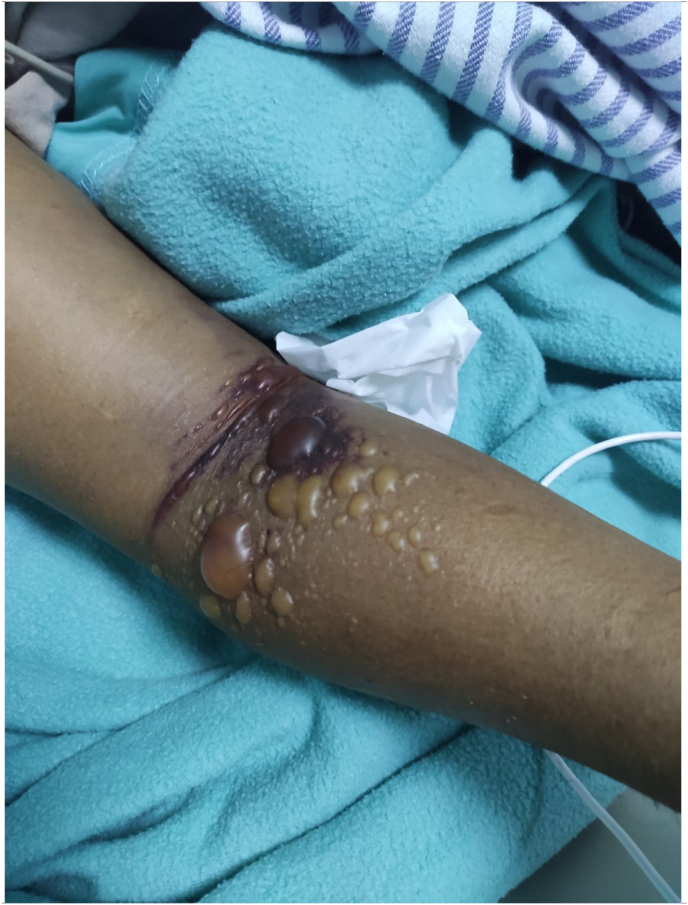
Bullae and echymosis during progression.

We perform laboratory work by blood drawing on the right cubital vein. Echocardiography showed normal kinetic with a preserved ejection fraction of 62%. He was further planned to undergo the early invasive strategy. However, within 2 hours of our care, there was significant swelling and tensing in his right (nontraumatic) brachial and antebrachial area (Fig. 3). In the next 2 hours, his swollen worsened into ecchymosis with bullae, and distal digital fingers developed pallor and paresthesia with decreased peripheral saturation of 88–91% (Fig. 4). We discontinue enoxaparin and proceed with single antiplatelet aspirin as recommended by ESC guidelines for NSTEACS 2020. He was then diagnosed with acute spontaneous compartment syndrome and planned to undergo fasciotomy.

**Fig. 5:**
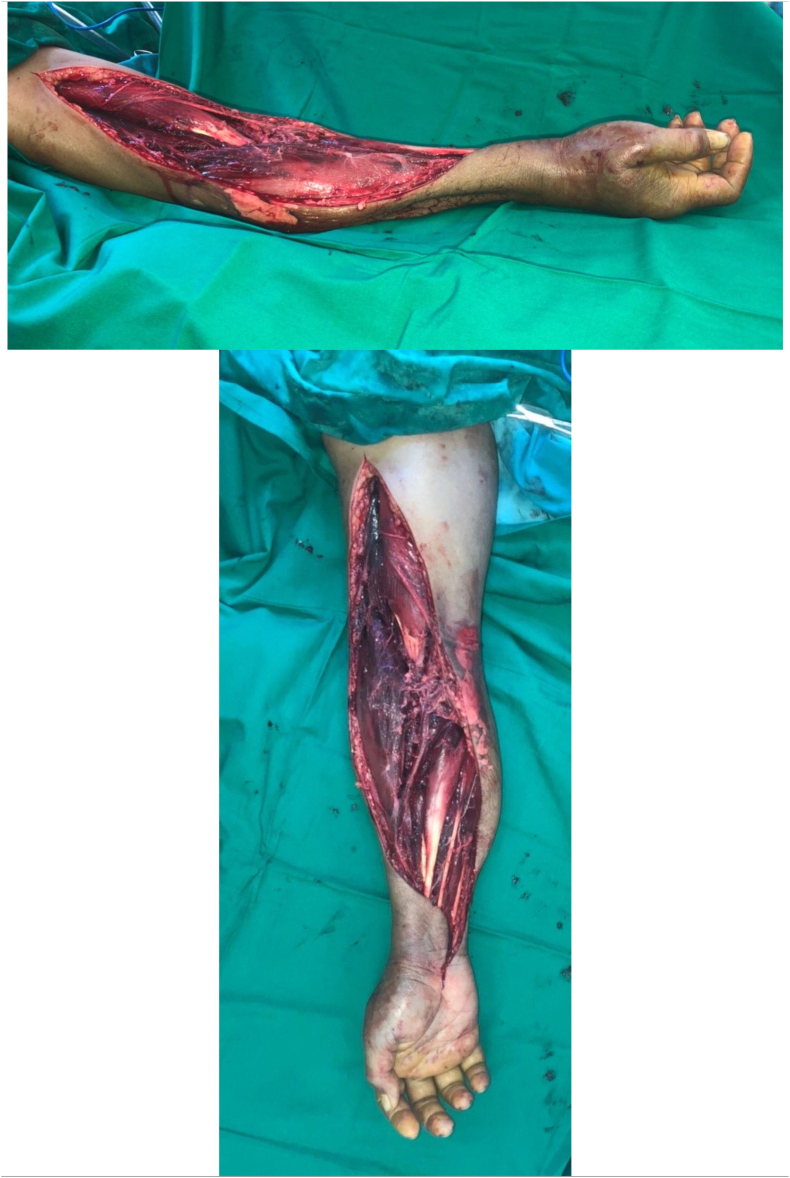
Post fasciotomy.

Postoperatively, his symptoms improve (Fig. 5). We proceed with a conservative strategy for his NSTEACS due to exposed fascia with active bleeding, which is a contraindication to antiplatelets if he was planned for coronary stenting.

## 3 Clinical discussion

Compartment syndrome is caused by markedly increased pressure through a closed space (compartment) that disrupts local circulation, bearing decreased perfusion within the compartment [2,3]. This condition is generally associated with risk factors, commonly comprising fractures, burns, venous obstruction, vascular injuries, or compressive casts as traumatic events [2,4]. Increased compartment pressure can be induced by either an increase in volume within a fixed compartment size or a decrease in compartment size. The increasing pressure first jeopardizes microcirculatory perfusion. Nonetheless, as the pressure rises, lymphatic, capillary, and tiny venule flow decreases, leading to compromise venous and arterial blood flow, resulting in tissue ischemia and necrosis. This disorder can lead to fibrous tissue deterioration, neurological impairment, contractures, and even amputation if not treated promptly. As vascular flow decreases, the pressure within the compartment rises, resulting in a vicious cycle of edema worsening [2,4,5].

The process shown by the aforementioned ailment is a vicious cycle of raising tissue pressure, causing ischemia, and irreversibly breaking down muscle. Because compartment syndrome is a clinical diagnosis, imaging approaches have limited diagnostic utility [4]. This illness's diagnosis is based on clinical judgments. Discomfort that was out of proportion to the damage, palpable swelling, pain on passive stretch test, focal motor or sensory deficits, and pulselessness were all present in the patient. Pulselessness and paralysis are late indicators of irreversible injury [2,4,5]. A diagnostic sensitivity of 93% is associated with the combination of pain with passive stretch, paresthesia, and pain at rest, with paresis boosting sensitivity to 98% [2].

Double-check data about the injury's history, such as risk factors and symptom changes, is crucial. Early indications and symptoms are often subtle, and only aware patients may detect them. The first sign is excruciating pain that is out of proportion to the test. Patients report the pain as intense, searing, and deep, which is increased by passive stretching. Pain is a subjective experience with little sensitivity, which is terrible. Side effects such as paresthesias, sensory abnormalities, and focal motor weakness are also possible [2].

Several case reports have described common causes of acute compartment syndrome without triggering traumatic events [3]. The mechanism of spontaneous acute compartment syndrome is unknown; however, a microvascular rupture in a patient with vasculopathy and high-risk bleeding (hemophilia, atypical localized infections, disseminated intravascular coagulation) induce gradual blood accumulation in the extremities compartment may cause acute compartment syndrome. Concurrent anticoagulation administration and all conditions impeding blood coagulation may increase the risk of this hypothetical pathophysiology [3]. As in our case, the patient was diagnosed with NSTEACS with impaired renal function (estimated glomerular filtration rate 7 ml/min/1.73m^2^) and wrongly received a twice-daily therapeutic dose of enoxaparin, a factor Xa inhibitor with a half-life of 7 hours in normal renal function. Enoxaparin was recommended to be administered with dosage modification in a patient with eGFR less than 30 ml/min. A calculated GRACE score of 57, as a parameter of bleeding risk in a patient with NSTEACS, further worsens the patient coagulation profile and adjoin his risk of developing spontaneous compartment syndrome. Atraumatic compartment syndrome, in which atraumatic exercise causes an increase in intramuscular pressure, has received just a few reports [4,5].

Only a few reports of nontraumatic acute compartment syndrome have been described in the literature. Atraumatic acute compartment syndrome is a difficult diagnosis to make because the triggering causes are unknown [6,7].

The arm comprises the anterior (biceps), posterior (triceps), and smaller deltoid compartments. Even though the anterior and posterior compartments can tolerate huge amounts of fluid, compartment syndrome can still develop. The ulnar and median nerves are located in the anterior compartment, as are the elbow flexor muscles. The radial nerve and the elbow's extensor muscles are both housed in the posterior compartment. The abductor muscles and axillary nerve are both housed in the deltoid compartment [1]. Deep volar compartment muscles (flexor pollicis longus and flexor digitorum profundus) and the median nerve are frequently damaged in the forearm compartments [6].

If the diagnosis of compartment syndrome is incorrect, major problems can occur, including severe muscular atrophy, loss of feeling, and even amputation. Because the clinical signal may not be clear in atraumatic situations, establishing a diagnosis might be difficult. A comprehensive history taking and physical examination must be accompanied by recognizing that nontraumatic compartment syndrome may be present [5]. When acute compartment syndrome of the upper extremities occurs for no apparent reason, the doctor should suspect an undetected bleeding condition [7].

This case depicts the development of events in a compartment syndrome that developed unexpectedly rather than as a result of a traumatic incident. This example demonstrates how anticoagulant usage can produce spontaneous bleeding, resulting in limb amputation (compartment syndrome) and perhaps death. Even if there is no history of trauma, patients with recognized risk factors and symptoms of acute compartment syndrome should be evaluated and consulted for surgery as soon as possible. In such cases, a strong suspicion, rapid diagnosis, and prompt surgery are the keys to avoiding serious consequences.

### 3.1 Strength and limitation

There are limited reports on the compartment syndrome of atraumatic origin in which nontraumatic event leads to an abnormal increase in intramuscular pressure. Atraumatic acute compartment syndrome is a challenging diagnosis as the precipitating elements are not widely known. These cases can very easily be neglected, and prompt treatment could be delayed.

## 4 Conclusion

Atraumatic acute compartment syndrome is an illness that requires prompt diagnosis and emergency treatment to avoid complications and preserve limb function. It is crucial to detect this condition to prevent postponing surgical surgery without a usual history of traumatic incident or underlying risk factors. Emergency decompression is recommended since a delay in diagnosis can result in long-term problems and the need for additional surgical treatments.

Clinical examination is still the most important despite current research on new diagnostic techniques. Early fasciotomy is the only effective treatment for preventing lasting impairments, which emphasizes the necessity of rapid diagnosis and action.

## Ethical approval

Not applicable.

## Sources of funding

None.

## Author contribution

All authors contributed toward data analysis, drafting and revising the paper, gave final approval of the version to be published and agree to be accountable for all aspects of the work.

## Research registration number

1. Name of the registry: Not applicable.

2. Unique Identifying number or registration ID:

3. Hyperlink to your specific registration (must be publicly accessible and will be checked):

## Guarantor

MA are the guarantor for this study.

## Consent

Written informed consent was obtained from the patient for the publication of the case report and the accompanying images. A copy of the written consent is available for review by the Editor-in-Chief of this journal on request.

## Declaration of competing interest

The authors have no conflicts of interest to declare.

## References

[1] Zimmerman D.C., Kapoor T., Elfond M., Scott P. (2013). Spontaneous compartment syndrome of the upper arm in a patient receiving anticoagulation therapy. J. Emerg. Med..

[2] Long B., Koyfman A., Gottlieb M. (2019). Evaluation and management of acute compartment syndrome in the emergency department. J. Emerg. Med..

[3] Via A.G., Oliva F., Spoliti M., Maffulli N. (2015). Acute compartment syndrome. Muscles. Ligaments Tendons J..

[4] How T.W., Irwan B.S. (2021). Atraumatic acute compartment syndrome in isolated medial compartment of foot, A rare case report. J. Biosci. Med..

[5] Cara J.A., Narváez A., Bertrand M.L., Guerado E. (1999). Acute atraumatic compartment syndrome in the leg. Int. Orthop..

[6] Chavez G., Choi J., Fogel N., Jaramillo J.D., Murphy M., Spain D. (2019). Atraumatic acute forearm compartment syndrome due to systemic heparin. Trauma Surg. Acute Care Open.

[7] Ogrodnik J. (2021). Clinical case of acute non-traumatic hand compartment syndrome and systematic review for the upper extremity. Hand.

[8] Agha R.A., Franchi T., Sohrabi C., Mathew G., Kerwan A., Thoma A., Beamish A.J., Noureldin A., Rao A., Vasudevan B., Challacombe B., Perakath B., Kirshtein B., Ekser B., Pramesh C.S., Laskin D.M., Machado-Aranda D., Miguel D., Pagano D., Millham F.H., Roy G., Kadioglu H., Nixon I.J., Mukherjee I., McCaul J.A., Chi-Yong Ngu J., Albrecht J., Rivas J.G., Raveendran K., Derbyshire L., Ather M.H., Thorat M.A., Valmasoni M., Bashashati M., Chalkoo M., Teo N.Z., Raison N., Muensterer O.J., Bradley P.J., Goel P., Pai P.S., Afifi R.Y., Rosin R.D., Coppola R., Klappenbach R., Wynn R., De Wilde R.L., Surani S., Giordano S., Massarut S., Raja S.G., Basu S., Enam S.A., Manning T.G., Cross T., Karanth V.K.L., Kasivisvanathan V., Mei Z. (2020). The SCARE 2020 guideline: updating consensus surgical CAse REport (SCARE) guidelines. Int. J. Surg..

